# The Impact of United States Medical Licensing Examination Step 1 Transitioning To Pass/Fail on Medical Student Perception of Research Needed to Match Into One's Preferred Specialty

**DOI:** 10.7759/cureus.57395

**Published:** 2024-04-01

**Authors:** Madisyn Currie, Carly Hammond, O. Parker Martinez, Abbi Lane-Cordova, James Cook

**Affiliations:** 1 School of Medicine, University of South Carolina, Columbia, USA; 2 School of Public Health, University of South Carolina, Columbia, USA; 3 Department of Obstetrics and Gynecology, Prisma Health Richland Hospital, Columbia, USA

**Keywords:** residency, board exam, pass fail, step 1, medical student

## Abstract

Purpose

To evaluate how the transition of United States Medical Licensing Examination (USMLE) Step 1 to a pass/fail scoring influenced medical student perceptions of the importance of research required to match into their preferred residency specialty.

Methods

A 14-item survey was distributed by e-mail to medical students at one medical school in the southeastern United States in November of 2021. Responses were compared between medical students taking USMLE Step 1 pass/fail in the future and medical students taking USMLE Step 1 for a three-digit score.

Results

A total of 168 medical students responded to the survey with 98 respondents who planned on taking USMLE Step 1 pass/fail (45 first-year medical students (MS1) and 53 MS2) and 70 respondents who took USMLE Step 1 for a numerical score (37 MS3 and 33 MS4). There were no differences in how each cohort scored the level of importance of research in matching into their preferred residency specialty (p=0.10); however, those taking USMLE Step 1 pass/fail believe an average of 4.6 research experiences are necessary to match into their preferred residency, compared to only 3.4 research experiences for those who took it for a numerical score (p=0.04).

Conclusion

No statistically significant difference in the perceived importance of research in matching into one’s preferred residency specialty was found between cohorts. However, the pass/fail cohort believes they will need more research experiences to match their chosen specialty than the numerical score cohort. Results could indicate that students participate in more research and extracurricular activities to be more competitive for residency applications.

## Introduction

On January 26th, 2022, the United States Medical Licensing Examination (USMLE) Step 1 transitioned to a pass/fail grading system instead of the traditional three-digit score [1]. This decision was ultimately made to decrease the emphasis on Step 1 scores in the match process and create a more holistic review process of applicants [2-4]. Step 1 scores were historically important for the match process as residency programs often used these standardized test scores to screen applicants for interviews because of the large number of students that apply for a limited number of residency positions [5,6]. This transition has created additional pressure on residency directors to find an objective standardized measurement to screen applicants for residency interviews. Many medical students and residency program directors are now contemplating to what degree this transition will impact students taking Step 1 in the pass/fail format and by what additional means program directors will screen upcoming residency applications for interviews.

The decision to transition to a pass/fail format may already be positively impacting medical students applying for residency. However, most of these impacts will not be immediately apparent, and there is currently little research on this topic from the students’ perspectives. Medical school deans have already stated that this transition will lessen the negative experience of studying for Step 1 on medical students' mental health and well-being as well as foster a more comprehensive method of looking at each medical student’s residency application [7]. An additional positive outcome is the speculation that this change to the scoring system could decrease disparities in cost and availability of Step 1 preparation materials among medical students [7,8].

Additionally, there are some negative outcomes of the transition of Step 1 to a pass/fail format. Some students may feel uncertain about how this change impacts them, leading to increased stress. The stress derived from the study process for Step 1 may now be heightened and transferred to the study process for Step 2, as program directors now rank Step 2 scores as having greater importance than previous application cycles [9-12]. Students may also feel further pressure to obtain an increased amount of research and involvement in extracurricular activities to distinguish themselves in the residency process. Moreover, this change may disproportionately affect medical students at lower-ranked medical schools, as medical school prestige may now be used as an objective measure to rank residency applicants [12]. Overall, this shift in the Step 1 scoring criteria may paradoxically create an increased burden of stress on medical students.

There is limited research on the transition of Step 1 to pass/fail and how it will impact medical students. Accordingly, this study aims to determine the relationship between the transition of Step 1 to a pass/fail format and the amount of research experiences students perceive as necessary to match into a chosen specialty. We predict that the students impacted by Step 1 transitioning to pass/fail will report needing a higher number of research experiences to match their specialty of choice compared to students who took Step 1 with a numerical score.

## Materials and methods

A cross-sectional study was performed by distributing a 14-item survey to all medical students, MS1-MS4, at one allopathic medical school in the southeast United States. The survey was generated through Google Forms and was distributed in November of 2021. Survey responses were coded and stored on Google Drive and could only be accessed by the research team. The data was exported into an Excel document and did not contain any identifying information. All students who responded to the survey were assumed to provide informed consent and were included in the study. Students excluded from the study included those less than 18 years old and those who were non-English speaking. Institutional review board (IRB) approval was obtained to distribute this survey.

Survey questions involved short answers, multiple choice, and Likert scale formats. The formats of survey questions were designed to allow students to rank how important research was to them, analyze student demographics, and obtain information on the quantity of research experiences.

The first section of the survey inquired about demographic information. This included gender identity, age, race, and year in medical school. The next section included questions about the student's academic background. This included earned degrees and medical school grade point average (GPA). The third section inquired about the first and second choices of residency specialty. The final section included questions about the importance of research for the match process, if Step 1 transitioning to pass/fail influenced the amount of research students were participating in and the number of research experiences to be competitive for the match process. The number of research experiences was a short answer question asking students to report their total number of abstracts, posters, publications, and presentations.

Students were divided into two cohorts. The pass/fail cohort included first- and second-year students (MS1 and MS2), who had not yet taken Step 1 and who would be affected by the transition to a pass/fail grading system. The numerical cohort included third- and fourth-year students (MS3 and MS4) who had already taken Step 1 and received a three-digit numerical score.

Data were analyzed using two-sample t-tests, Pearson correlation coefficients, and Fisher’s exact tests to compare responses between cohorts. Data from survey responses are demonstrated in Tables 1-2. When the cohorts being analyzed by two-sample t-tests resulted in unequal variances, Satterthwaite methods were used to correct this. All statistical analyses were performed using Stata 17.0 (StataCorp, College Station, TX).

**Table 1:** Student Demographic Data Two individuals did not report gender. One individual did not report age. Four individuals did not report race. Three individuals did not report GPA. GPA: grade point average; OB-GYN: obstetrician-gynecologist; SD: standard deviation.

	Numerical Cohort (n=70)	Pass/Fail Cohort (n=98)	P value
Gender			0.346
Female	30	50	
Male	39	47	
Mean Age (SD)	24.6 (3.1)	26.6 (2.9)	<0.001
Race			0.460
American Indian or Alaska Native	0	0	
Asian	9	13	
Black or African American	1	1	
Hispanic or Latino	2	0	
Native Hawaiian or Other Pacific Islander	0	0	
White	56	82	
GPA			0.382
4.0-3.5	48	55	
3.49-3.0	18	33	
2.99-2.5	3	7	
2.49-2.0	0	1	
Degree Attainment			0.661
Undergraduate Degree	61	82	
Additional Graduate Degree	9	16	
Preferred Residency Specialty			0.061
Internal Medicine	14	10	
Surgery/Surgical Subspecialty	13	37	
OB-GYN	7	10	
Pediatrics	6	11	
Psychiatry	6	3	
Emergency Medicine	7	11	
Family Medicine	3	4	
Neurology	0	2	
Other	14	10	

**Table 2:** Medical Student Perceptions on the Importance of Research to Match into Preferred Specialty and Mean Perceived Number of Research Experiences Necessary to Match These perceptions are measured on a Likert scale ranked from one to five. Five is very important to match, and one is not important.

	Numerical Cohort (n=70)	Pass/Fail Cohort (n=98)	P value
Research importance ranking (1-5)			0.102
1	3	3	
2	4	6	
3	21	16	
4	25	32	
5	17	41	
Mean perceived research experiences needed (SD)	3.4 (3.1)	4.6 (4.2)	0.042

## Results

A total of 370 medical students from a single public medical school in the southeastern United States were asked to complete the survey. Out of these 370 students, 168 (45.4%) students completed the survey. Table 1 describes the characteristics of respondents. Of those that responded, 98 students (45 first-year and 53 second-year medical students) will be taking USMLE Step 1 as pass/fail, and 70 students (37 third-year and 33 fourth-year medical students) have already taken USMLE Step 1 for a numerical score. There was no significant difference in gender, race, GPA, postgraduate degree attainment, or preferred residency specialty between the pass/fail cohort and the numerical score cohort. The numerical score cohort was older with an average age of 26.6, compared to an average age of 24.6 in the pass/fail cohort (p<0.001). For the cohort that is taking USMLE Step 1 pass/fail, 80 (81.6%) responded “yes” to expect to participate in a greater amount of research now that the scoring format for Step 1 has changed.

Table 2 reports the results describing medical students' perception of research experiences needed to match their preferred specialty. When asked to rank the importance of research on a scale of one to five, with five signifying the highest importance, 41 (41.8%) of those in the pass/fail cohort ranked importance as a five, while only 17 (24.2%) of those in the numerical score cohort ranked importance as a five. However, there was no significant difference in how each cohort ranked the importance of research (p=0.10). The cohorts did differ in how many research experiences were believed essential to match into a preferred residency specialty. The pass/fail cohort reported an average of 4.6 experiences while the numerical score cohort reported an average of 3.4 experiences (p=0.04). For students in both cohorts, a higher ranking of research importance correlated with a larger number of research experiences deemed necessary to match one's preferred specialty (p<0.001).

## Discussion

The conversion of the USMLE Step 1 exam from a three-digit numerical score to a pass/fail score is a decision that has raised many questions and differing opinions among medical students, residency directors, and others involved in medical school education. There is speculation on what will be the focus of residency directors in separating medical student applications in the match process. Some predict that Step 2 scores, standardized clerkship National Board of Medical Examiners (NBME) scores, and subjective clerkship evaluations may take the place of Step 1 numerical scores [13]. However, the focus of this study was to analyze medical students’ perception of the importance of research on the residency match process. The data from this study was analyzed to ascertain whether a transition in score reporting will create additional pressure for involvement in an increased amount of research experiences to become more competitive in the residency match process. 

To our knowledge, this study is one of the first to analyze the score report change from a medical student’s perspective, specifically relating to the emphasis on the quality and quantity of research. The only significant difference in demographic factors between the two cohorts in this study was an increased average age in the cohort that completed Step 1 for a numerical score. This was to be expected given that this cohort is farther along in their medical school career than the cohort that will be taking Step 1 for a pass/fail score. In this study, there were no significant differences between the cohorts on preferred residency specialty. However, it has been reported in the literature that attitudes toward pass/fail scoring are strongly correlated with interest in highly competitive specialties. It is expected that the change in score reporting will affect students matching into highly competitive specialties differently than it will affect students matching into less-competitive specialties [14]. There will no longer be the invariable emphasis placed on Step 1 numerical score as a first-line measure for residency programs, which has been met with some opposition from students interested in highly competitive specialties [6,11]. This creates the need for other metrics to identify students in the application process, most presumably with an emphasis on research [11,15].

When both cohorts were asked to rank the importance of research throughout the medical school for the match process, there was no significant difference between the two groups. This highlights the integral element of research productivity that is well established in the match process, regardless of the recent change in Step 1 score reporting [15]. In this survey, we determined that of those medical students taking Step 1 for a pass/fail score, 81.7% of them expected there to be an increase in the number of research experiences they participated in compared to if Step 1 scoring was numerical. Furthermore, although there was no difference in research importance between the two cohorts, there was a statistically significant difference in the number of research experiences expected to match a preferred residency specialty between the two cohorts. This highlights our proposed emphasis that research involvement will be represented in the match process for students with a pass/fail score on Step 1.

It is anticipated that less time will be spent on Step 1 preparedness. This is considered an advantage of the pass/fail score reporting that allows for increased student well-being and additional time for students to strengthen their residency application with extracurricular opportunities, such as research experience and community involvement [16]. The drawback to this decision is that students attending medical schools that are considered lower-tier programs and/or have a less-established research infrastructure might be negatively affected [14]. We predict that unless further developments and opportunities are created in research at all medical doctor (MD), doctor of osteopathic medicine (DO), and International medical graduate (IMG) programs, the stress burden of studying for Step 1 will only be transferred to the task of obtaining involvement in research experiences.

The limitations of our study should be noted. This survey was distributed to medical students at one medical school in the southeastern US that has a focus on primary care. We do not claim to represent all medical students and chose to highlight only one aspect resulting from the Step 1 pass/fail score reporting. The external validity of this study’s cohort and results may not be generalizable to students at other allopathic medical schools in the US, osteopathic medical schools in the US, or international medical schools as a result. Responses from this survey were collected and analyzed by students who attended the medical school where the survey was distributed. As a result, some of the information students may have provided on the survey could be attributed to social desirability response bias. If this survey was completely anonymous, this bias may have been reduced. Second, some of the questions on the survey were not considered to be straightforward. One short answer survey question instructed students to put the number of research experiences thought to be necessary to match into their chosen specialty. Most of the respondents answered this short answer question with a range of experiences perceived as necessary to match instead of a precise number. Out of the 168 students that responded, there were 47 students that put a range of values. As a result, the lower number of the reported range was incorporated into the data to exclude information bias. This suggests that the results may be lower for this variable than in reality. Additionally, there are more residency specialty options that could have been listed on the survey. There were 24 students out of the 168 students that chose “other” as their answer choice, so the results may have been influenced by this extraneous variable. Finally, another limitation of this study is that the numerical score cohort might have had additional access to advising that could have impacted survey responses. Residency programs also start offering interviews during September for residency positions. This survey was distributed in November, and, by this time, some fourth-year students may have had interviews leading to more confidence in their ability to match. As a result, this may have led students to report a lower number of research experiences to match their chosen specialty compared to the pass/fail cohort. All things considered with the limitations listed, this study carefully investigated the dependent variable of research relating to Step 1 pass/fail score reporting in this cohort of medical students.

## Conclusions

In summary, the transition of the USMLE Step 1 from a numerical score to a pass/fail score reporting will affect many aspects of medical student education and the residency selection process. The findings in this study represent the perspectives of US medical students on the well-known role of research experience in the match process. Students who will be taking Step 1 pass/fail reported a higher average volume of research experiences required to match into a residency specialty of choice when compared to students who took Step 1 for a numerical score. More investigation and feedback are required to understand the effects, including, but not limited to, increased medical student research in the wake of the transition of Step 1 score reporting and the influence it will have on medical student well-being.
